# A Possible Role of Intestinal Microbiota in the Pathogenesis of Ankylosing Spondylitis

**DOI:** 10.3390/ijms17122126

**Published:** 2016-12-17

**Authors:** Lianjun Yang, Liping Wang, Xin Wang, Cory J. Xian, Hai Lu

**Affiliations:** 1 Academy of Orthopedics of Guangdong Province, Orthopaedic Hospital of Guangdong Province, Department of Orthopedic Surgery, The Third Affiliated Hospital of Southern Medical University, Guangzhou 510630, China; lianjunyang1988@163.com (L.Y.); liping.wang@mymail.unisa.edu.au (L.W.); 2 Sansom Institute for Health Research and School of Pharmacy and Medical Sciences, University of South Australia, Adelaide SA5001, Australia; 3 Institute of Health and Biomedical Innovation, Queensland University of Technology, Brisbane QLD4059, Australia; x56.wang@hdr.qut.edu.au

**Keywords:** ankylosing spondylitis, intestinal, microbiota, microbiome, treatment

## Abstract

Ankylosing spondylitis (AS) is a chronic inflammatory disease primarily affecting the sacroiliac joints and the spine, for which the pathogenesis is thought to be a result of the combination of host genetic factors and environmental triggers. However, the precise factors that determine one’s susceptibility to AS remain to be unraveled. With 100 trillion bacteria residing in the mammalian gut having established a symbiotic relation with their host influencing many aspects of host metabolism, physiology, and immunity, a growing body of evidence suggests that intestinal microbiota may play an important role in AS. Several mechanisms have been suggested to explain the potential role of the microbiome in the etiology of AS, such as alterations of intestinal permeability, stimulation of immune responses, and molecular mimicry. In this review, the existing evidence for the involvement of the microbiome in AS pathogenesis was discussed and the potential of intestinal microbiome-targeting strategies in the prevention and treatment of AS was evaluated.

## 1. Introduction

Ankylosing spondylitis (AS) is a chronic inflammatory disease which mainly involves the sacroiliac joints and spine; it is considered to be most likely due to the interaction of genetic factors and environmental factors, such as microbial triggers [1,2,3]. Its prevalence currently ranges from 0.10% to 1.60% in the Europe [4]. The disease is often found among those 15 to 30 years of age and is characterized by inflammatory back pain particularly at the sacroiliac and spinal joints and by progressive ankylosis and stiffness.

AS is the primary form of seronegative spondyloarthropathies (SpAs) which include AS, psoriatic arthritis, reactive arthritis, and arthropathy of inflammatory bowel disease (IBD). The mechanisms leading to AS are unknown, although human leukocyte antigen B27 (HLA-B27) is known a major risk factor for AS [5]. HLA-B27 is known a genetic marker present in 90%–95% of AS patients [6]. However, genetic predisposition fails to fully explain the cause of the disease, and this leads to a strong effort to identify additional predisposing factors.

Many studies support the notion that the gut microbiota plays an important role in AS, although its precise mechanism has not yet been fully understood. Advances in the technology of next-generation sequencing and bioinformatics tools will facilitate the characterization of the human microbiome and will likely provide information on the early events of AS pathogenesis. Future research will differentiate the possible direct and indirect effects of the composition of gut microbiota on the incidence of AS and the progression of the disease. In this review article, available data from animal and clinical studies on the potential relationship of gut microbiota with AS will be reviewed, focusing on roles of the microbiome in AS and identifying potential novel therapeutic strategies for preventing or treating AS by targeting microbiota which should hamper the role played by gut microbiota dysbiosis in the development of AS.

## 2. Evidence of AS-Microbiome Interactions

In animal studies, HLA-B27 transgenic rats do not develop many features of SpAs when raised in a germ-free environment [7]. However, arthritis develops when commensal bacteria, such as *Bacteroides vulgatus*, are introduced into these germfree models [8]. HLA-B27 transgenic rats after transfer to a conventional rat colony appear with symptoms similar to AS. These studies indicate the role of the microbiome and genetic susceptibility in the initiation of inflammatory response.

Oral microbiota (with over 700 bacterial species being harbored within the oral cavity) have a role in periodontal disease and, in turn, play a contributory role in periodontitis. It has also been reported that periodontitis is linked to AS, and patients with AS are more likely to suffer periodontitis [9]. Comparing AS patients with healthy people, AS patients have the higher level of anti-*Pophyromonas gingivalis* [10], again suggesting potential interaction between some specific microbiota with AS.

Despite the unusual high AS heritability, the twin concordance rate is not 100%. The microbial environment may play a role in an HLA-B27 genetic background [11,12]. The role of infectious microbial triggers in AS, however, is controversial. With the rapid development of high-throughput technology, there is a deeper understanding on the composition and function of the human microbiome [13,14,15]. An interesting report by Martínez et al. suggested infection was closely related with patients with SpA. Their research shows that 56 percent of patients had infections: including upper respiratory infection, urinary system infection, and intestinal infections. Moreover, HLA-B27 positive patients have a higher infection rate, and the intestines are the most commonly involved [16].

Some bacteria have been reported to play an important role in AS pathogenesis, such as *Klebsiella pneumoniae* and *Bacteroides vulgatus* [17]. However, the amount of bacteria usually involves causal immune response instead of infection. A number of studies have in fact demonstrated that *Klebsiella pneumoniae* play a role in the pathogenesis of the disease. Ebringer et al. demonstrated that AS was strongly associated with the fecal presence of *Klebsiella pneumoniae* when the disease was in an active inflammatory state [18]. In a similar study, the presence of fecal *Klebsiella aerogenes* was also found to be significantly associated with peripheral synovitis in HLA-B27 positive patients [19]. Furthermore, some studies also indicated that *K. pneumoniae* antibody was closely related to intestinal inflammation in patients with axial form of AS [20,21]. However, other studies did not find a similar phenomenon [22]. Stone et al. [1] did not find a causal relationship between *K. pneumoniae* and AS. These contradictory results were probably due to operation error or patients being at different stages [23].

## 3. Roles of the Intestinal Microbiome in AS

There are trillions of bacteria reside within the human intestine, which constitute the complex microbiota system known as the gut microbiome and play a critical role in intestinal health [24]. The intestinal flora disturbance can lead to lots of diseases, including diabetes, obesity, chronic kidney disease, and IBD [25,26,27,28]. About 70 percent of patients with AS have subclinical gut inflammation, which indicates that the two diseases may be similar entities with a common origin, that is, gut dysbiosis [29,30,31]. Recently, it has been shown that the occurrence of spondyloarthritis is influenced by the intestinal microbiota [32,33,34,35]. The role of gut microbiome in AS pathogenesis is also suggested by many studies [29,36,37], and AS can be associated with IBD [38,39]. During the active stage of the disease, most of the patients exhibited an elevated serum total IgA level, suggesting microbial translocation and intestinal barrier failure [40]. A recent study has indicated that, in the terminal ileum and when compared to healthy controls, AS patients had a higher abundance of five bacterial families (e.g., *Lachnospiraceae*, *Prevotellaceae*, *Rikenellaceae*, *Porphyromonadaceae*, and *Bacteroidaceae*) and a lower abundance of two bacterial families (*Ruminococcaceae* and *Rikenellaceae*) [29]. An interesting report by Tito et al. suggested that the status of intestinal inflammation associated strongly with the profile of mucosal microbiota of SpA patients and that Dialister could be as a potential microbial biomarker of SpA disease activity [33]. Stebbings et al. [41] demonstrated a higher fecal presence of sulphate-reducing bacteria in AS patients, which have been implicated in the IBD pathogenesis. Furthermore, it was also noticed that breastfeeding, which induces a different microbiota from the one induced by bottle feeding, could prevent the development of AS. Montoya et al. [42] found evidence that breast feeding protects against the development of AS. They think that the influence of breast feeding on AS development may involve direct interaction of human milk proteins or lipids with the infant’s immune system, or indirect interaction involving the infant’s gut flora.

Evidence from animal models indicates that, when raised in a germfree environment, HLA-B27 transgenic rats fail to develop inflammatory peripheral joint or intestinal disease. However, upon receiving transfer of the common gut bacteria *bacteroides*, the disease occurred [7]. It has also been shown that, in colonized mice, the luminal anaerobic bacteria triggered ankylosing enthesopathy, and that joint inflammation was prevented in mice raised in germ-free conditions [43]. Interestingly, Rath et al. [8] demonstrated that, in HLA-B27 transgenic rats, six commensal bacteria—including *Bacteroides vulgatus*—could cause colitis and gastritis. Furthermore, the results of another study have shown that the fecal samples of HLA-B27 and human β2-microglobulin (HLA-B27/hβ2m) transgenic rats had an increased presence of *Prevotella* spp. but a decreased abundance in *Rikenellaceae* when compared to the wild type rats. Their results indicated that altered fecal microbiota is associated with HLA-B27 [44], and that intestinal microbiota are critically important in the development of chronic intestinal inflammation [45].

Taken together, multiple lines of evidence firmly implicate intestinal microbiome as a possible contributor to the pathogenesis of AS. Therefore, characterization of the species composition of microbiota associated with AS and the exact mechanism for the functional role of intestinal microbiome in disease progression should be the focus of research in the near future.

## 4. How the Gut Microbiome Affects AS

**Figure 1:**
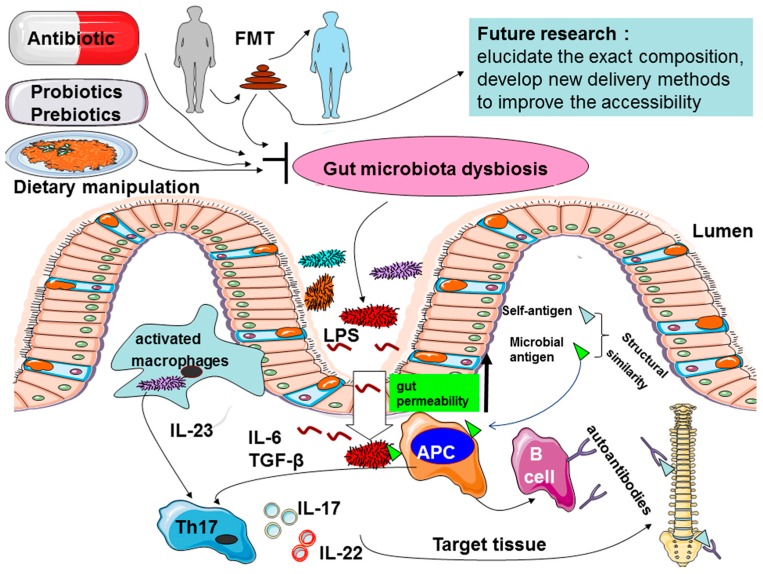
An overview of the possible mechanisms for gut microbiota dysbiosis in causing inflammation in ankylosing spondylitis (AS) and potential targeting alternative therapy strategies aiming to reduce the microbiota dysbiosis. Abbreviations: antigen-presenting cell (APC); B cell: B lymphocyte; fecal microbial transplantation (FMT); T helper cells (Th cells); lipopolysaccharide (LPS).

While evidence for the role of the gut microbiota in the AS progression is mounting, several mechanisms have been suggested to explain the role of microbiome in the etiology of AS, such as alterations of intestinal permeability, stimulation of immune responses, and molecular mimicry (Figure 1). There is crosstalk between the gut microbiota and the immune system when gut microbiota disturbance occurs [13].

### 4.1. Increased Intestinal Permeability

The intestinal epithelium is an important physical and biochemical barrier against commensal and pathogenic microorganisms, which conserves host–microbial interactions and maintains tissue homeostasis [46]. When a problem in the competency of the tight junctions (connections between epithelial cells) occurs, it will lead to increased intestinal permeability, which is called a leaky gut. Dysbiosis of the intestinal microbiota can lead to damage the mucosal barrier and increase penetration of commensal microbiota, which are important in the process of disease occurrence and development [47,48,49].

Gut permeability has been found to have increased in AS patients and their first-degree relatives as well as in experimental animal models, which perhaps allows a greater exposure systemically to gut microbes [50]. Steven et al. showed that gut permeability in HLA-B27 rats was nearly five times higher than in healthy control rats, and it also increased with age [51]. The changes of intestinal permeability may occur before intestinal inflammation [52]. Lipopolysaccharide (LPS), a toxic part of endotoxin, will induce a serious systemic inflammatory response if entering the bloodstream. Some preliminary evidence has suggested that high serum levels of LPS and fatty acid binding protein can be present in AS, which is significantly associated with gut permeability [46].

### 4.2. Increased Gut-Joint Axis of Inflammation through Regulating Innate Immunity and IL-23/Th17 Axis

#### 4.2.1. Innate Immunity

The innate immune system, also known as in-born non-specific immunity system, is the first line of defense that protects the host body from invasion of pathogens. During this innate immune response process, intestinal macrophages play a central role in protecting intestinal barrier function and against commensal. Receptors of macrophages can recognize attack invaders, like bacteria or viruses [31,53,54,55,56,57]. Several reports have indicated that dysfunction of intestinal macrophages may increase the incidence of IBD [58,59,60,61], and bacteria can induce macrophage differentiate into different types of macrophage with different function. Macrophages are divided into two types: M1 or M2 [62]. M1 macrophages produce relatively higher levels of pro-inflammatory cytokines, including tumor necrosis factor α (TNFα) and interleukin-12 (IL-12), whereas M2 macrophages suppress inflammation in numerous disorders [63].

The increase of M1 macrophages was observed in AS and Crohn’s disease (CD) patients with intestinal inflammation. Meanwhile, M2 macrophages were also expanded in AS patients. However, some AS patients did not display excessive intestinal inflammation although there was an increased presence of M1 polarized macrophages. One possibility is that the significant increase in M2 macrophages could offset the pro-inflammatory of M1 macrophages [64]. In another study, in peripheral spondyloarthritis (SpA), Vandooren et al. found an increased expression of M2 markers CD163 and CD 200R and a decrease in proinflammatory mediators derived from M1, including TNFα and IL-1β [63]. Interestingly, another study demonstrated that the level of IL-23 produced by subchondral bone marrow M2 macrophages from AS patients was significantly increased when compared to normal control specimens [65]. However, despite the above studies, representations of different subsets of macrophages are still unclear in the AS patients, and it is possible that the acute and chronical intestine tissue inflammation states of AS patients may represent different pathologic disease situations.

#### 4.2.2. IL-23/Th17 Axis

There is evidence that the pattern of cytokine secretion influences the pathogenesis of AS and gut immunity. Alteration in the interleukin-23 (IL-23)/Th17 signaling axis has been suggested as an important trigger for chronic inflammation [66,67,68,69,70,71,72]. Briefly, this pathway corresponds to the polarization of naïve CD4 positive cells towards a T-helper cell (Th17) phenotype, associated with by the predominant production of IL-17 and IL-22. This differentiation is caused by IL-23 (and also IL-6 and TGF-β) realizing the IL-23/Th17 pathway [73].

Expression of IL-17 and IL-23 has been found increased in peripheral blood, gut, and skeletal tissue [74,75]. While IL-17 and IL-23 may be produced by the gut, mechanisms how the microbiome affects action of IL-23/IL-17-producing cells are still being investigated. It is postulated that microbial antigens may lead to an increased expression of cytokines, such as IL23 and IL-17 [76]. For example, IL-23 can be triggered by *Chlamydia trachomatis* which is related to the reactive arthritis [77]. In another study, *Salmonella enteritidis* was found to induce local Th17 responses in animal reactive arthritis models [78]. In humans with SpA, innate lymphoid cells expressing IL-23R were found able to migrate from the gut to peripheral blood, bone marrow, and joints [79]. In the gut, the microbiota dysbiosis is able to induce the rapid release of IL17 from innate-like immune cells [36,80].

IL-23, in particular, has recently been suggested as a key cytokine involved in regulating innate and adaptive immune responses in AS [81,82,83,84,85]. In patients with AS, IL-23 was found to express in cells within the subchondral bone marrow and in some macrophages within fibrous tissue replacing the bone marrow in facet joints [65]. In addition, Ciccia et al. indicated that infiltrating monocytes were responsible for the increased IL-23 expression and overexpression of IL-23 is an important characteristic of subclinical gut inflammation in AS [66]. IL-23, IL17, and IL-22, have been suggested to be involved in the gut-joint axis of inflammation [86]. IL-17 exhibits proinflammatory properties which may result osteoclastogenesis and inflammatory bone loss. Nevertheless, IL22 may lead to bone outer layer soft tissue swelling as is seen in periostitis and AS [87]. In curdlan-treated ZAP-70W163C mutant BALB/c (SKG) mice, IL-23 could result in arthritis, enthesitis, and ileitis. IL-22 affects different tissues. On the one hand, IL-22 could result in osteitis, On the other hand , it plays a protective effect on ileum. IL-17 is associated with enthesitis [88]. A recent study indicated that patients with AS had an elevated frequency of IL-17A^+^ mucosal-associated invariant T (MAIT) cells and there was an enrichment of MAIT cells in the synovial fluid. Meanwhile, IL-17 elevation in AS MAIT cells was dependent on priming with IL-7 stimulation [89,90]. Previously, MAIT cells have been implicated in a variety of autoimmune diseases, such as multiple sclerosis, IBD, and rheumatoid arthritis (RA) [91,92]. Additionally, MAIT cells are critically dependent on the gut for their development, and this strengthens the role of IL-17 and the concept of a gut-joint axis of inflammation in AS.

Taken together, these studies suggest that aberrant IL-23/Th17 immune response activation is an important feature in AS patients and targeting the IL-23/Th17 response pathway could likely be a target for treatment of AS patients [93,94].

### 4.3. “Molecular Mimicry” or “Cross-Reactivity”

Molecular mimicry is defined as sequence or structural similarities between foreign antigens and self-antigens [95]. These foreign antigens, mostly from invading microbes, activate immune cells to produce antibodies. These pathogen-specific antibodies not only react with microbes but also bind to host self-antigens, which will subsequently result in inflammation and tissue damage. Molecular mimicry or cross-reactivity hypothesis is another mechanism which could trigger AS [96], while molecular mimicry is the major mechanism of rheumatic fever [97].

HLA-B27 itself may cross-react with Gram-negative bacteria. For example, *Klebsiella* microbes possess various antigens which show molecular similarity and immunological cross-reactivity with HLA-B27 or other self-antigens. A homology of six amino acids has been found between HLA-B27.1 antigen (residues 72–77) and *Klebsiella pneumoniae* nitrogenase (residues 188–193) [98]. It has also been shown that the Pul-D secretion protein “DRDE” sequence (residues 596–599) of *Klebsiella* pullulanase enzyme is similar to the “DRED” motif (residues 74–77) of HLA-B27 [99]. Antigenic extracts of five gut-inhabited bacterial agents—such as *Klebsiella*, *Enterobacter*, *Salmonella*, *Shigella*, and *Yersinia* microbes—showed positive reactions with antibodies from a rabbit immunized with HLA-B27-positive lymphocytes indicating the presence of shared cross-reactive antigens [100]. *Klebsiella* can stimulate host to produce different types of across-reactive antibodies, and each type will target specific part of the body, especially those high expression of HLA-B27 antigen [101,102]. Furthermore, there is also molecular mimicry between *Klebsiella* pullulanase A and collagens (types I, III, and IV), thereby providing a possible explanation for the spinal locations of the pathological lesions in AS [99]. Thus anti-*Klebsiella* antibodies are acting potentially as autoantibodies against HLA-B27 and spinal collagens and therefore AS can be considered as an autoimmune disease.

Although many studies indicate the microbiome is associated with AS, the underlying mechanisms remain unknown. Thus further study is needed.

## 5. Intestinal Microbiome Targeting Strategies

The goals of AS treatment are to reduce symptoms, improve/maintain normal posture and spinal flexibility, maintain the ability to work, reduce functional limitations, and to limit AS-associated complications. The current AS medical treatments act by suppressing symptoms and are not specific, and these include the use of non-steroidal anti-inflammatory drugs, immunosuppressive drugs, and biological agents [103]. As changes in gut microbiota are an important factor in the pathogenesis of AS, they can be considered a novel therapeutic target for AS. As such, an alternative therapy for this medical predicament can be developed, which should be harmless and aimed at the elimination of the most plausible microbial agent, such as *Klebsiella*, in order to improve or even halt the inflammatory and pathological damage occurring in patients with AS. Therefore, further discussion is merited on these microbiota-based potential treatments as a potential therapy for AS patients (Figure 1).

### 5.1. Antibiotics

It is well known that antibiotics can dramatically decrease the biodiversity of the gut microbiota after prolonged use. Manipulation of luminal content of the gut using antibiotics may represent a potentially effective therapeutic option.

Sulfasalazine, which is a combination of salicylate (the main ingredient in aspirin) and a sulfa antibiotic, has an effect in the acute exacerbations of AS [104,105,106]. Alteration of bowel flora can be one of the mechanisms by which sulfasalazine exerts its therapeutic effect. In a randomized, multicenter trial, sulfasalazine improved peripheral joint disease, but no effect was observed on axial symptoms [107].

Because of the relationship of AS to certain Gram-negative bacteria, several clinical studies have assessed the utility of Moxifloxacin to treat AS. Moxifloxacin is a fluoroquinolone antibiotic with activities against both Gram-positive and -negative bacteria. The results of a similar study have shown that AS patients treated with Moxifloxacin resulted in a significant and sustained improvement and the mean of erythrocyte sedimentation rate and C-reaction protein decreased significantly after 12 weeks [108]. It was also noticed that some South African plant extracts with *K. pneumoniae* inhibitory activity can potentially prevent the AS onset and minimize the symptoms of the established disease [109].

Taken together, it is possible that the use of antibiotics could reduce the bacterial loads in the bowel of AS patients and prevent further tissue damage.

### 5.2. Probiotics and Prebiotics

Probiotics are defined as live microorganisms, which colonize the gut and promote benefits to host health [110,111,112,113,114,115]. Prebiotics can modulate the structure and metabolism of beneficial microbes in the colon and improve host health [116,117,118,119]. Proposed beneficial effects of probiotics and/or prebiotics include modulation of intestinal microbiota, strengthening of the epithelial barrier, and immunomodulation [120,121]. As indicated from evidence below, modulating intestinal microflora with probiotics or prebiotics could potentially be used as a supplement in addition to antimicrobial measures in patients with AS.

Amdekar et al. [122] showed that *Lactobacillus casei*, as a probiotic, has potent anti-inflammatory and antiarthritic effect. In their study, *Lactobacillus casei* have significant preventive effect on collagen-induced arthritis rat model which shown normal histopathology and low expression of proinflammatory cytokines. They think the possible mechanism of the phenomenon is *Lactobacillus casei* could alleviate the damage caused by prostaglandins. Another study demonstrated that oral administration of probiotic species *Lactobacillus rhamnosus GG* (L GG) can partially prevent relapse of colitis in antibiotic-treated specific pathogen free HLA-B27 transgenic rats [123]. Furthermore, a prebiotic (Synergy 1, which is a preferential inulin-oligofructose growth substrate for the probiotic strain), can decrease the incidence rate of colitis in HLA-B27 transgenic rats. Synergy 1-treated rats had shown an increased proportion of beneficial bacteria in the cecum and decreased pro-inflammatory cytokines, such as IL-1 [45].

Yet, evidence to support the use of probiotics as a treatment for these diseases is still intriguing and inconclusive. In a randomized controlled trial, 63 patients with active spondyloarthritis were randomly divided into two groups: 32 patients received probiotic while the other 31 patients received placebo. At the end of the study, there were no significant differences between the two groups [124]. In a similar study, 147 patients with spondyloarthropathy were enrolled into internet-based randomized controlled trials. Probiotic or placebo was given to volunteers to examine whether probiotic can relieve symptoms of spondyloarthropathy. Their study concluded that probiotics worked no better than a placebo [125]. There are four possible principal explanations for these findings: probiotics may have no effect on spondyloarthritis, a longer duration of therapy may be required, or probiotics may show benefits in early stages of SpA, and a beneficial outcome may require a larger dose or different mixture of strains of probiotics.

The above evidence suggests that probiotics and prebiotics could be a potential and appealing therapeutic strategy for AS; however, further interventional studies are still needed for investigating their treatment efficacies and exploring how host microbiome and probiotics or prebiotics interact.

### 5.3. Dietary Manipulation

Although the composition of the colonic microbiota in adults is relatively stable, its concentration, however, can be manipulated by dietary means [126,127]. For example, it has been shown that a high intake of carbohydrates, particularly oligosaccharides, can stimulate the growth of *Bifidobacterium* spp., *Klebsiella* spp., *Clostridium* spp., and *Escherichia coli* in the human colon [128]. Furthermore, high-energy diets have been shown to facilitate absorption of bacterial lipopolysaccharide (LPS) from intestinal bacteria [129]. Several reports have indicated that a low-starch diet has potential mechanisms of benefit for AS. Starch are broken down into simple sugars which are essential to the growth of gut microbes [103]. Most of the intestinal bacteria take part in the process of fermentation of starch [130]. Therefore, cutting down starch intake may benefit patients with AS.

There was a study which suggested that 36 active AS patients benefit from low starch diet. After nine months of treatment, the symptoms relieved in most of these AS patients, and some patients indicated that their requirement of medication also decreased [40]. Ebringer et al. demonstrated that in 36 patients with active AS receiving a low-starch diet, there was a significant drop in the erythrocyte sedimentation rate, total serum IgA, and their anti-inflammatory medicine intakes at the nine-month follow-up. They also reported that the mean number of *Klebsiella* microbes was 10 times higher for three different sugars (glucose, sucrose, and lactose) per gram of substrate compared to the number obtained from incubation with 11 different amino acids, thus showing that carbohydrates are highly necessary for the growth and replications of *Klebsiella* or even other related enteropathics [131,132].

Therefore, it is plausible that a drop in the starch intake in daily dietary consumption in conjunction with the use of the current treatment could be beneficial in managing patients with AS, thereby reducing inflammation and leading to clinical remission.

### 5.4. Fecal Microbial Transplantation

Fecal microbial transplantation (FMT) is the stool transfer from a healthy donor to restore a stable microbial community of the gut in a recipient [77,133,134,135]. Several studies indicate that FMT has a curative effect in treating recurrent *Clostridium difficile* infection and it has also been used in the treatment of many kinds of disorders, including IBD, diabetes, allergic diseases, neurological diseases, and obesity [136,137,138,139,140,141,142,143]. In a recent study, 19 patients with CD were treated with FMT and all patients completed the 12-week study period. The clinical symptoms of most patients were relieved and the gut microflora diversities of recipients were significantly higher compared with those before treatment [144]. In a systematic review, 41 patients with IBD treated with FMT showed a reduction or complete resolution of symptoms in 76% patients [145]. Another study demonstrated that FMT improves the quality of life significantly in patients with IBD patients [146]. The possible mechanism of FMT is to restore a normal intestinal microbiome and regulate the immune homeostasis of recipient [137]. Interestingly, a recent study published in 2015 did not report results consistent with the above. This recent study [147] showed no significant differences in endoscopic and clinical remission between Ulcerative Colitis (UC) patients receiving healthy donor fecal microbiota and those receiving their own fecal microbiota. Clearly, larger studies are urgently needed to further establish the efficacy of FMT.

However, FMT has not been evaluated in AS and more evidence is needed. Despite its enormous potential, there are some major challenges in using fecal microbial therapy. On the one hand, donors could unwittingly pass on an infection to the patient through stool donation [148]. On the other hand, there are many unanswered questions regarding the best route of administering the FMT. Further studies are required to elucidate the exact composition of the ecosystem being administered which may thus enhance the overall safety of this approach. Furthermore, there is a need to develop new delivery methods to improve the accessibility. Although there are still many areas of uncertainty concerning this emerging technology, it is plausible that the field of microbial manipulation in AS via FMT remains ripe for exploration.

## 6. Conclusions

The pathogenesis of AS is thought to be the result of a combination of host genetic and environmental triggers. Since highly plausible links exist between indigenous gut microbes and AS, the human gut microbiome represents an important area for etiological discovery. Several mechanisms have been suggested to explain the role of microbiome in the etiology of AS, such as alterations of intestinal permeability, stimulation of immune responses, molecular mimicry, and so on. However, none of these fully explain the etiology of AS and the search for possible causes continues. In the last decade, mapping the human microbiome has received increased attention [149]. Molecular-based methods, such as metagenomics, have allowed for more comprehensive and precise identification of the species composition of microbiota compared to traditional culture-based methods. However, many challenges still exist for meaningful discovery in this field.

The major challenges now remain to confirm the role of microbiota in AS, and to investigate how the microbiome shapes the immune response and influences both local and systemic inflammation. These answers will potentially reveal important insights into early events in the pathogenesis of AS. Meanwhile, further studies are needed to identify high-risk patients early, to accurately identify patients who have a poor prognosis and to commence treatment without delay. Low-starch diets, antibiotics, probiotics, prebiotic, and fecal microbial transplantation warrant further exploration as potential future therapeutic strategies for AS. These therapies may help to avoid the irreversible sequelae of established AS and in generally helping the sufferers to reduce the severity of their symptoms. In addition, identifying patients who would benefit from these therapies will also be clinically important.
